# “I don't like to be seen by a male provider”: health workers’ strike, economic, and sociocultural reasons for home birth in settings with free maternal healthcare in Nigeria

**DOI:** 10.1093/inthealth/ihac064

**Published:** 2022-09-27

**Authors:** Anthony Idowu Ajayi, Bright Opoku Ahinkorah, Abdul-Aziz Seidu

**Affiliations:** Sexual, Reproductive, Maternal, New-born, Child and Adolescent Health (SRMNCAH) Unit, African Population and Health Research Center, Manga Close, Nairobi, Kenya; School of Public Health, Faculty of Health, University of Technology Sydney, Sydney, NSW, Australia; College of Public Health, Medical and Veterinary Sciences, James Cook University, Australia; Centre for Gender and Advocacy, Takoradi Technical University, Takoradi, Ghana

**Keywords:** health facility, health system, health workers strike, home-based child delivery, maternal health, maternal mortality, sociocultural norms, transportation

## Abstract

**Background:**

Ending maternal mortality has been a significant global health priority for decades. Many sub-Saharan African countries introduced user fee removal policies to attain this goal and ensure universal access to health facility delivery. However, many women in Nigeria continue to deliver at home. We examined the reasons for home birth in settings with free maternal healthcare in Southwestern and North Central Nigeria.

**Methods:**

We adopted a fully mixed, sequential, equal-status design. For the quantitative study, we drew data from 211 women who reported giving birth at home from a survey of 1227 women of reproductive age who gave birth in the 5 y before the survey. The qualitative study involved six focus group discussions and 68 in-depth interviews. Data generated through the interviews were coded and subjected to inductive thematic analysis, while descriptive statistics were used to analyse the quantitative data.

**Results:**

Women faced several barriers that limited their use of skilled birth attendants. These barriers operate at multiple levels and could be grouped as economic, sociocultural and health facility–related factors. Despite the user fee removal policy, lack of transportation, birth unpreparedness and lack of money pushed women to give birth at home. Also, sociocultural reasons such as hospital delivery not being deemed necessary in the community, women not wanting to be seen by male health workers, husbands not motivated and husbands’ disapproval hindered the use of health facilities for childbirth.

**Conclusions:**

This study has demonstrated that free healthcare does not guarantee universal access to healthcare. Interventions, especially in the Nasarawa state of Nigeria, should focus on the education of mothers on the importance of health facility–based delivery and birth preparedness.

## Introduction

For decades, ending maternal mortality has remained an important global health priority.^1^ Reducing maternal mortality by 75% between 1990 and 2015 and universal access to reproductive healthcare services were key targets of the Millennium Development Goals (MDGs).^1^ Many sub-Saharan African (SSA) countries implemented user fee removal as one of the main interventions to achieve the MDG targets.^2–10^ However, only a few countries achieved the target, prompting it to remain one of the critical targets of Sustainable Development Goal 3, which aims to reduce the global maternal mortality rate to <70 per 100 000 live births by 2030. Efforts to achieve this target have prioritised SSA,^11^ where 66% of global maternal deaths occur.^12^

Several SSA countries, including Burkina Faso, Ghana, Niger, Kenya, Burundi, Mali, Uganda and Senegal, have adopted policies that remove or substantially reduce user fees for maternal healthcare services.^2–10^ The main goal of this policy is to ensure universal access to facility-based delivery,^13,14^ given that quality, skill-based delivery is considered the cheapest way to prevent maternal deaths. However, studies have reported mixed effects of this policy on maternal healthcare services utilisation. While a few studies show that user fee removal has enhanced equitable access to healthcare for pregnant mothers,^15–17^ a few studies did not report significant effects.^18,19^ Evidence shows that the quality of the free maternal healthcare policy is affected by several challenges, including delays in reimbursing health facilities, overcrowding and prolonged waiting time.^10,20–23^ These challenges have resulted in women bypassing health facilities offering free services to access care in those that do not grant user fee removal.^21–23^ The introduction of free maternal healthcare policy exacerbates inadequate resources, making some services unavailable.^22,24^

Nigeria has a huge burden of maternal mortality. The user fee removal policy was adopted in 2006 to reduce maternal deaths.^25^ However, maternal mortality rates in the country have seen only a slight improvement. Evidence shows that Nigeria's maternal mortality rate (MMR) is 512 maternal deaths per 100 000 live births in 2018.^26^ One in 22 women in the country is at risk of dying during pregnancy, childbirth, post-partum or post-abortion.^12^ The high MMR in the country has been attributed to several factors, including inaccessibility of health facility deliveries.^13,27^ Despite introducing the user fee removal policy, a huge proportion of women continue to give birth at home,^26^ suggesting that barriers other than user fees hinder women from accessing health facility delivery.

Studies elsewhere have identified several challenges with free maternal healthcare that have resulted in women choosing home-based deliveries. Such challenges include a lack of transportation to health facilities, cultural beliefs, lack of family support and poor quality of care.^28–30^ Despite these available evidence, studies that have explored the reasons for the use of home-based delivery amidst free maternal healthcare in Nigeria are scanty.^27,31–33^ Under the subreinvestment program, free maternal healthcare services were implemented in many Nigerian states between 2009 and 2015. Evaluation of the programme found varying effects on health facility–based delivery, with significant improvement recorded in some states^34^ while no progress was recorded in other states.^13,14,35^ What is evident is that many women continue to give birth at home despite Nigeria's free maternal healthcare policy.^13,14^ However, we found no studies examining why women still choose home-based childbirth in settings with user fee removal. Evidence on why women continue to give birth at home in this context is important for policymakers to re-evaluate the programme and tailor their interventions to reach all women. In this article, we drew on data from a mixed-methods study among women who gave birth at home to understand the reasons for this in the context of the user fee removal policy.

## Methods

### Study setting, design and population

The data analysed in the current study were drawn from a large cross-sectional study from the MANCONFREE project, which sought to examine maternal outcomes in the context of free maternal healthcare in three Nigerian states (Ekiti, Nasawara and Ondo). This research was conducted in three states chosen purposively from two of Nigeria's six geopolitical zones. These states were selected because of the distinctiveness of their free maternal health programs. Maternal healthcare policies differ from state to state, different approaches to implementing healthcare policies. Ondo and Ekiti were chosen in the Southwestern geopolitical zone, while Nasarawa was chosen in the North Central. The programme's implementation in the specified study locations has been published in previous studies.^13,14,36^ In Ekiti State, free healthcare was implemented only in selected primary healthcare facilities, but it was implemented in all state government–owned health facilities in Ondo and Nasarawa States. According to the 2013 Nigeria Demographic and Health Survey report, the proportion of births in health facilities was 86.3% in Ekiti State, 56.2% in Ondo State and 40.1% in Nasarawa.^37^

The study was conducted between May and September 2016. The study adopted a fully mixed, sequential, equal-status design involving a survey of 1227 women of reproductive age who gave birth in the 5 y before the survey, six focus group discussions and 68 in-depth interviews. The quantitative data analysed in this article are limited to the structured responses of 211 women in our survey who gave birth at home.

Two-stage cluster random sampling was used to select participants from their households in both rural and urban enumeration areas. The sample size was sufficient for the main study. The qualitative component of the study used a purposive sampling technique to recruit participants. Participants who met the inclusion criteria were approached face to face by the research team and asked to participate in the interviews voluntarily. They mostly represent a diverse group, including young and old and from urban and rural households. The characteristics of the participants of the in-depth interviews (IDIs) and focus group discussions (FGDs) are published elsewhere.^36^ The data were collected with the help of research assistants who were well trained on how to use the instruments and research ethics. The IDIs took an average of 50 min to complete, while the FGDs took approximately 1.5 h. The interviews were conducted in English, Yoruba and Hausa in participants’ homes at their convenient times. All the interviews were audio recorded and field notes were also taken. The IDIs and FGDs conducted in the local languages were translated into English by bilingual translators. Translations were validated by the first author, who is fluent in Yoruba, and research assistants fluent in the Hausa language. Participants granted permission for the interviews to be audio recorded.

Our analysis in this article is limited to transcripts covering reasons for home delivery emanating from the interviews. The trustworthiness of the qualitative data was ensured using the following guidelines proposed by Lincoln and Guba.^38^ These are credibility, transferability, dependability and confirmability. The credibility of the data was ensured by carefully selecting experienced field assistants who were trained before the data collection. All the interviews were audio recorded and field notes were taken to capture the non-verbal cues during the interview process. Our use of the purposive sampling technique, which allowed the selection of information-rich participants who met the inclusion criteria, helped guarantee credibility. Thorough explanations of the research methods that include essential details that a reader would need to know to understand the results helped ensure transferability. We ensured dependability by using an emergent design analysis where new issues surfaced throughout the succeeding data gathering and analysis phase. The involvement of multiple researchers in the data collection phase guaranteed that data interpretations arose through researcher triangulation, increasing the trustworthiness of the data source. The first author, who was involved in data gathering and supervision, ensured that all the research assistants adhered to all the ethical principles and the collection of quality data. All transcribed audios were verified to ensure no meaning was lost throughout the translation process as part of the member checking procedure, which was utilised to collect the data and satisfy the confirmability criterion.

### Data analyses

We used descriptive statistics to analyse the quantitative data from 211 survey participants who gave birth at home. We stratified reasons for home delivery by state to illustrate how they vary by study setting. The qualitative data were translated and transcribed. All transcripts were edited to remove grammatical errors while maintaining the original meaning. We used inductive thematic analysis^39^ to analyse the transcripts related to reasons for home delivery. The first author was already familiar with the data, given that he conducted about 40 of the 68 interviews and four of the six FGDs. But to better comprehend the data, we read the transcripts and the field notes for familiarisation and immersion.^40^ The data coding followed iterative and collaborative processes, with the first author generating the initial codes and the research team discussing, reviewing and codeveloping the final codes. This ensured analytical rigour, transparency and trustworthiness. We then grouped the codes under themes that reflect the study objectives. Supporting quotes were presented when appropriate in the manuscript to illustrate pertinent themes. The social constructionist theory notion that meanings are developed in coordination with others rather than individually informed our analysis and interpretation.^41^ It guided us to seek shared reasons from the respondents on why they prefer home-based child delivery despite user fee removal programmes.

## Results

**Figure 1.**
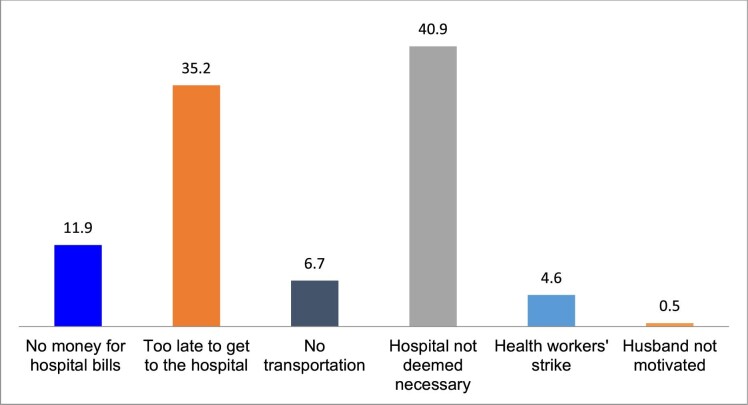
Reasons for giving birth at home.

Exploring why women give birth at home is critical to understanding maternal health-seeking behaviours in the context of free maternal healthcare. Our analysis draws on both quantitative and qualitative data. Convergence and parallels were drawn from the two sources of data. It must be reiterated that the goal of mixed study design in this study is not to prioritize the findings of a method, but rather to use a method to complement the other. Equal priority is therefore given to each method. As presented in Figure 1, lack of money, birth unpreparedness, lack of transportation, strikes, unsupportive husbands and perceived non-necessity of skilled birth attendants were the main reasons for giving birth at home. However, birth unpreparedness (35.2%) and perceived non-necessity of skilled birth attendants (40.9%) were the most cited reasons by the study participants.

**Figure 2.**
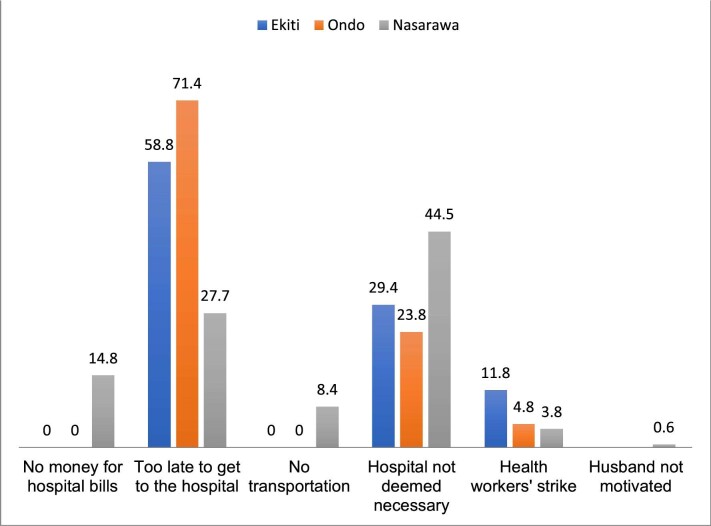
Reasons for giving birth at home: analysis by states.

The reasons given for giving birth at home vary by state. While the lack of money and transportation were some of the reasons for giving birth at home in Nasarawa State, they were not in Ekiti and Ondo States. As shown in Figure 2, health workers strikes were reported in all three states. However, birth unpreparedness was the main reason for giving birth at home in Ondo and Ekiti States (Figure 2).

**Figure 3.**
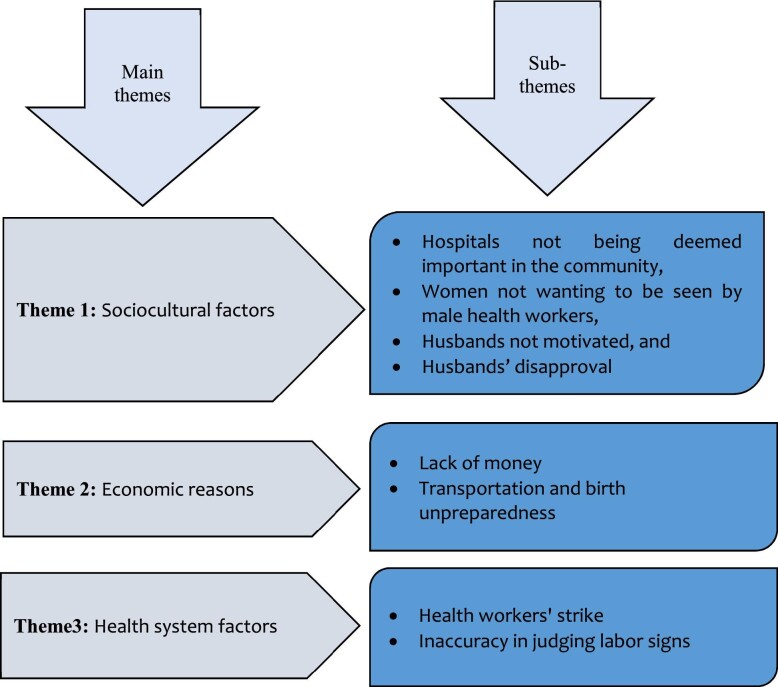
Main themes and subthemes.

The findings from the qualitative study shed some light on the quantitative results. The findings of the qualitative study are presented in Figure 3.

### Sociocultural factors

Despite the introduction of the user fee removal policy, a few women favoured home delivery for sociocultural reasons. These sociocultural reasons include hospital delivery not deemed necessary in the community, women not wanting to be seen by male health workers, husbands not motivated and husbands’ disapproval. Participants who believe giving birth in skilled facilities is unnecessary often consider giving birth to be very easy and home-based childbirth customary. The interview with a teenage mother of two from a rural area of Nasarawa who gave birth all by herself in her home explains the perceived non-necessity of skilled delivery. She was unfazed by childbirth and proud to give birth to her son alone. She explained that most women give birth at home, and it is customary to give birth at home even though they often attend antenatal care in a close-by village. She gave birth to her two children at home and only attended antenatal care twice. Although uneducated, her decision to give birth at home seems related to customs around childbirth rather than her education level. Nonetheless, none of the interviewees from urban areas considered childbirth in skilled delivery facilities unnecessary.

In most interviews and FDGs in rural communities lacking health facilities in Nasarawa State, childbirth is deemed not to require a hospital visit. This view was supported by a 30-year-old mother of four:

‘…childbirth does not require health facility visit. Most women deliver at home in our village, and we are expected to deliver at home. When I had my last child, I just closed the door and delivered alone’ (FGD participant 29, Nasarawa State, 30 July 2016)

She responded proudly, suggesting that they take pride in being able to deliver without support. While home delivery is customary in the rural villages of Nasarawa lacking health facilities, it appears that the lack of health facilities has sustained the cultural practices of home delivery.

Although only two participants reported that they prefer to give birth at home because they do not like to be seen or touched by a male health worker, this contributes to why women do not use skilled birth facilities. The two participants had varied reasons for not wanting to be touched by a male health worker. The first woman, a 45-year-old mother of six children, attended antenatal care but gave birth to all her six children at home due to her dislike for being examined by a male doctor. She was quick to point this out when asked why she gave birth at home:

I use public hospitals for antenatal care, but I gave birth to all my children at home. I wouldn't say I like hospitals or men's presence while giving birth. (In-depth interview participant 62, Ondo State, 27 July 2016)

Culturally, childbirth is a women's affair and men are not expected to be in the labour room. However, some male midwives and doctors attend deliveries in health facilities, contrary to her cultural values. She stated that no man other than her husband should have to see her nakedness and men do not have to witness the delivery. Since she did not want to risk a male health worker attending her delivery, she chose home delivery for all her six children.

The second woman believed that being touched by doctors or nurses would make her too weak to deliver by herself. Surprisingly, this woman had a higher degree, although she resides in a rural area. It could be that her community's culture prevailed over her education and she is looking for ways to justify her choice of home birth. She indeed justifies her belief by stating:

I prefer to give birth at home because I do not want to be touched by nurses and doctors. Being touched several times would weaken me to the extent that I may not have the strength to deliver the baby when it is time. (In-depth interview participant 51, Nasarawa State, 5 August 2016)

Other sociocultural reasons for home delivery in the settings with user fee removal policies were husbands not motivated and outright disapproval. While this finding is less common in Ekiti and Ondo, a few women in Nasarawa alluded to husbands’ disapproval as a barrier to health facility delivery. As expressed by the women, the husband's lack of motivation means they offered no financial and material provisions to facilitate hospital delivery even though they did not expressly stop their wives. It also could mean they were unenthusiastic about facility-based delivery. However, there are few cases where husbands expressly disapproved of health facility–based delivery. When pressed on the reason she gave birth at home, a 27-year-old mother of three said:

I wanted to deliver in a health centre, but my husband refused. (In-depth interview participant 51, Nasarawa State, 5 August 2016)

### Economic factors

Other than sociocultural factors, economic factors still present a barrier to health facility delivery in the context of free maternal healthcare services. Surprisingly, some participants mentioned the lack of money as one reason for the non-utilisation of skilled birth attendants despite the availability of free maternal healthcare. However, the consensus from the FDGs is that childbirth is not completely free, as one still has to buy some delivery items, such as baby dresses with woollen caps, gloves, stockings, soaps, oil, methylated spirit, hot water flask, delivery mat, towels, breastfeeding bras, wipes, jik bleach or hypo, cord clamp, baby sponge, hand gloves, cotton wool, diapers and a shawl. A woman in her mid-30s echoed this during the group discussion:

Childbirth in health facilities is not entirely free. You still have to pay to register. You also pay for baby materials. In fact, you are given a list of things you must buy. Sometimes they reject some of the items you bought and you are told to buy the same item from the hospital. (FGD participant 14, Ondo State, 28 July 2016)

Health workers often prescribe a list of things to buy, and the amount required to buy these items depends on an individual's financial capability. Nonetheless, the backlash women receive from health workers and other women for not purchasing them or not buying adequate items hinders them from utilising skilled birth attendants. One middle-aged woman (44 y) pointed out:

Giving birth in the hospital with incomplete delivery items and baby clothing is like ridiculing oneself. The nurses will mistreat you, and even other women will look at you disdainfully. (In-depth interview participant 2, Ekiti State, 2 July 2016)

Women who cannot afford baby materials are believed to be treated differently by health workers. An overzealous nurse who does not see from the poor pregnant woman's perspective sometimes lessens the individual's sense of pride to the extent that some women prefer to give birth at home to avoid being ridiculed. A mother of four children who had first-hand experience of being ridiculed by nurses corroborated this assertion in the in-depth interview:

I prefer to give birth at home because I cannot afford to buy delivery items like other women. I do not particularly appreciate how the nurses berated me during my first child's birth just because I did not come with all the items on the list given to me. (In-depth interview participant 8, Ekiti State, 8 July 2016)

Some participants simply could not afford the user fees charged at the hospital for childbirth. In rural Nasarawa, most women confirmed that there was no free maternal healthcare, even though this is not the case in Ondo and Ekiti States. Evidently, women residing in Nasarawa were more likely to state a lack of money was the reason for their non-utilization of the skilled birth facility.

Transportation costs and unavailability hinder women from using skilled birth attendants, especially in rural Nasarawa. Unlike Ekiti and Ondo States, many women in Nasarawa still reside >20 km from facilities. This makes the cost of transportation a financial burden to the family despite user fee removal. Women expressed this during the FGD in Nasarawa State:

The health centre is far away from our community. Sometimes I did not go for antenatal care because we could not afford the cost of transportation. I did not even bother to go to the health centre for delivery because we could not afford it. (FGD participant 33, Nasarawa State, 4 August 2016)

Even women who can afford the cost of transportation sometimes are unable to find appropriate means of transportation to convey them to the health facility. A 30-year-old woman who had four children shared:

We go for antenatal care in the clinic in this next village, but difficulties in finding transport during delivery make us deliver at home. (FGD participant 31, Nasarawa State, 30 July 2016)

This challenge is often exacerbated when labour starts at night and no healthcare facility is nearby. The problem of transportation is not limited to rural areas and is often related to birth unpreparedness. Indeed, women residing in the city do face transportation challenges primarily due to birth unpreparedness. Most women who reported that it was too late to get to the hospital meant that the time between labour and delivery was insufficient for them to arrange transportation. A middle-aged woman who had previously given birth to two children in a skilled birth facility but experienced home birth during the index pregnancy noted:

The delivery happened at night, and the baby came quickly, even before my husband could arrange for a means of transportation. (In-depth interview participant 12, Ekiti State, 10 July 2016)

Most families depend on public transportation because their families do not own private cars. In many underserved communities, car ownership is very low and the few people who own cars use them for public transport.

### Health system factors

Although the user fee removal policy has addressed the supply side of access to care, it has not completely addressed health system factors. Health workers’ strikes were frequent and women expressed concerns about providers’ ability to accurately judge labour signs in primary healthcare facilities. As recounted by the study participants, public health facilities’ inaccessibility due to health workers’ strikes contributes to why women give birth at home. Recently, health workers’ strikes have become incessant in Nigeria, negatively affecting users of government-owned skilled birth facilities. Voices from the IDIs and FGDs echoed the harmful and damaging consequences of the health workers’ strikes. A middle-aged woman whose friend died due to post-partum haemorrhage after giving birth at home opined that her friend would still be alive today had the health workers not been on strike:

She was bleeding after childbirth at home, and we rushed her to the hospital, but there was no doctor to attend to her. She passed before we could access a doctor in a private facility as a last resort.

Many women registered in public health facilities would either turn to private practice or give birth at home. Of course, the charges in private health facilities are more than those of government facilities.^42,43^

Another health system factor responsible for why some women deliver at home, as pointed out during the interviews, is the inability of health workers to accurately judge the timing of childbirth in primary health centres. Some women reported that they were told they were not yet ready to give birth, only for them to return home and deliver shortly after that. A woman, who has a higher degree, experienced this and wants the health workers to be trained on how to accurately judge labour signs:

I went to the hospital for my son's birth but was told I was not ready to give birth, so I went home, only to get home, and the baby came. So, I think they should teach the health workers how to know when a woman is ready to give birth. (In-depth interview participant 62, Nasarawa State, 3 August 2016)

## Discussion

Free maternal healthcare was introduced to eliminate financial barriers preventing women from accessing health facility delivery.^14^ Despite this, some women in settings with user fee removal programmes still give birth at home. Therefore, this study sought to understand why home-based delivery continues even though the policy is meant to change this. We found that individual, sociocultural and health facility–related factors influenced the decision of women to give birth at home despite the free maternal healthcare policy. The primary reasons were lack of money, birth unpreparedness, lack of transportation, unsupportive husband, perceived non-necessity of a skilled birth attendant and health workers’ strikes. However, perceived non-necessity of skilled-based child delivery and birth unpreparedness were the most cited reasons by the study participants. Our analysis shows that the reasons for giving birth at home varied by state. While the lack of money and transportation were reasons for giving birth at home in Nasarawa State, they were not in Ekiti and Ondo States. Health workers’ strikes were reported in all three states. However, birth unpreparedness was the main reason for giving birth at home in Ondo and Ekiti States. It is worth noting that from 2010 to 2015, the policy was fully implemented in all government-owned health facilities in Ondo State but in selected government-owned health facilities in Ekiti and Nasarawa States.^14^

Our study shows that the user fee removal policy did not wholly eliminate financial barriers to health facility delivery. Costs beyond user fees exist and hinder women from accessing skilled delivery in settings with user fee removal policies. These costs include transportation and delivery and baby items. Consistent with previous studies,^28,30^ lack of money was among the reasons women delivered at home. However, eliminating financial barriers and increasing access to and use of skilled delivery services were the goals of the free maternal healthcare policy.^34^ That notwithstanding, there are still some indirect costs, such as unavailable drugs and the cost of delivery kits. Consistent with our findings, Boah et al.^44^ found that difficulties in acquiring items (such as sanitary pads, disinfectants, napkins, clothes for the baby and drugs) pushed women to choose home-based child delivery despite Ghana's free maternal healthcare policy. Women who cannot afford these items feel embarrassed and even reported being ill-treated by some medical personnel because they did not have them. Also, lack of transportation was previously reported as one of the reasons for not seeking antenatal care in Nigeria.^45^ Lack of transportation constitutes a barrier to using maternal care services in other SSA settings, as reported in previous studies.^28,30,46^ Long distance from health facilities and unavailability of transportation prevent women from accessing maternal health services, including health facility delivery. Labour can occur anytime, and finding a means of transport is challenging in underserved communities, forcing women to deliver at home in such settings.

Ajayi and Akpan^13^ explained that in settings where user fees are removed, the uneven distribution of health facilities creates a barrier to using maternal healthcare services. Many low-income women living in underserved areas live far away from health facilities, causing them to travel considerable distances to access free maternal healthcare. For the poor, transportation costs often account for a significant portion of their household income. Poor women who reside in underserved areas do not always benefit from the user fee reduction policy compared with women of the middle and high socio-economic classes.^13^ This is also discussed within the health services utilization model by Anderson and Newman,^47,48^ which indicates that factors such as long distance to health facilities and unavailability of means of transport are disabling factors to the use of health services. In addition, the three-delays model by Thaddeus and Maine^49^ has also espoused that inaccessibility to health facilities in the form of transportation is one of the reasons for delays in seeking healthcare, including delivery services.

The sociocultural reasons for home delivery include hospitals not being deemed important, women not wanting to be seen by male health workers, husbands not motivated and husbands’ disapproval. A lack of perceived need for skilled health facility delivery can be discussed within the context of low health literacy. Several previous studies have reported this.^28,30,44^ Women's and partners’ awareness and understanding of maternal health issues improve when exposed to maternal health information. Abebe et al.^50^ reported that women who gave birth at home lacked a sense of the importance of maternal health services. Poor and less educated women have benefited from health education and promotion efforts.^51^ Our findings on partners’ disapproval, not being motivated and women not wanting to be attended to by male healthcare workers are similar to what has been reported in previous literature.^44,52^ Husbands’ involvement in helping or limiting wives’ access to facility-based delivery is complex and changes depending on the context. Sometimes a spouse can be a facilitator by persuading his wife to visit a facility and arranging transportation and cash. On the other hand, a husband may refuse his wife's request to visit the health facility due to financial or cultural reasons.^53^ Therefore, it is imperative to increase health education for women and their partners to counter some sociocultural beliefs that serve as barriers to health facility delivery.

Another important finding in our study was health system factors that comprised health workers’ strikes and inaccuracy in judging labour signs by nurses. All these hinder access to health facility delivery. Thaddeus and Maine's three-delay model^49^ refers to delays in deciding to seek care, arriving at a health facility and receiving acceptable care. The third delay—the time it takes to receive care after arriving at a health facility—indicates a problem with healthcare delivery.^30^ Healthcare workers’ strikes have varied negative implications on health delivery. According to the study, many women registered at public health facilities would have to either go to a private clinic or give birth at home. Of course, private healthcare facilities charge more than government-run facilities. The findings suggest that health workers’ strikes significantly impact pregnant women's health-seeking behaviour. Most of those who said they had signed up for antenatal treatment in ‘unaffordable’ private maternal health facilities said they were compelled to do so by (fear of) public-sector labour strikes. Evidence suggests that in 2017, the public health system was near collapse due to the health workers’ strike in Kenya.^54^ This reduced hospital admissions and outpatient services, including skilled delivery.^55^ Poor women are most affected by health workers’ strikes since they are least likely to afford care in private health facilities.^54^ Despite the damaging effects of health workers’ strikes on maternal outcomes, they have become far too familiar in Nigeria since 2014. Resident doctors were on strike in Nigeria in 2021 when we drafted this manuscript, indicating that this has become a pervasive problem with dire implications for women and girls’ well-being. Women who were unable to raise the user fees in private hospitals opted for giving birth at home. Overall, the analysis shows that pregnant women's health-seeking behaviours were greatly influenced by perceived or real health workers’ strikes. Most participants stated that they registered for antenatal care in both public and private health facilities due to anticipated health workers’ strikes in the public health centres. However, private health facilities come at huge costs, too much to bear for some women, who thus opted for birth at home. Participants agreed that health workers’ strikes are the leading cause of maternal deaths and attributed strikes as the reason their friends died due to childbirth complications during a recent health worker's strike in Nigeria.

## Strengths and limitations of the study

The study obtained data from diverse settings with three implementation sites. The data were also collected from diverse groups with different cultural and socio-economic backgrounds. That notwithstanding, the study had the following limitations. First, the study could not obtain nurses’ views on the inability to judge labour signs accurately. It would be worth getting the opinions of health workers on the reasons why they return the women home despite their conviction that they are due for delivery. Also, the study did not capture the views of the men who played key roles in decision making regarding choosing the place of delivery.

## Conclusions

This study has demonstrated that free maternal healthcare services do not guarantee universal healthcare access. Many women will continue to give birth at home until we can permanently solve issues of healthcare providers’ strikes, change socio-cultural norms hindering facility-based delivery and empower women in diverse ways, including financial empowerment. Thus, it is paramount to improve health education to make women and their partners appreciate the importance of maternal health services use, males’ involvement in maternal healthcare, adoption of various means to satisfy the demands of health workers to avoid strikes and the need for health workers to do a critical assessment of the stages of dilation in labour to avoid misinforming women who present to health centres.

## Data Availability

Data will be made available by the corresponding author upon reasonable request.
